# Supplementary material for: Timely Association of RSV Hospitalisation Waves in Children with the Detection of SARS-CoV-2 in the General Population in Eastern Bavaria
Source: Viruses. 2025 Dec 5;17(12):1584. doi: 10.3390/v17121584 (PMC12737403; doi:10.3390/v17121584)
Supplement: Supplementary file 1 [file viruses-17-01584-s001.zip › viruses-3873868-supplementary.docx]

**Syntax forecast.linear excel:**

forecast.linear(x,known_y’s,known_x’s): estimated hospitalisation

x: month for which the hospitalisation rate was calculated

known_y’s: hospitalisations for each month in the low prevalence seasons 2017/2018, 2019/2020 , and 2021/2022

known_x’s: months in which the hospitalisations in 2017/2018, 2019/2020 , and 2021/2022 occurred

The equitation for forecast.linear is a + bx, where:

$$a= \bar{y}-b\bar{x}$$

and

$$b= \frac{\sum\left( x-\bar{x} \right)\left( y-\bar{y} \right)}{{\sum\left( x-\bar{x} \right)}^{2}}$$

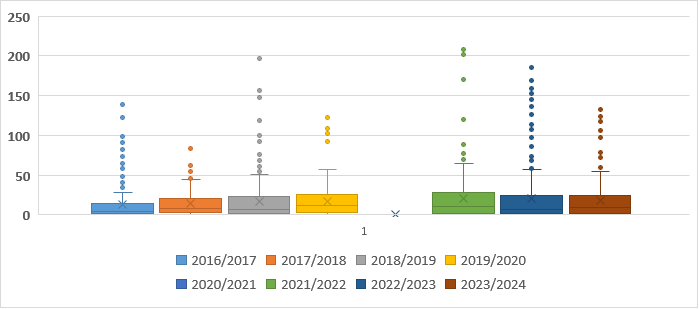


**Supplemental** **Figure S1: Age of hospitalised RSV patients (Regensburg) in months**

**Supplemental** **Figure S2: RSV hospitalisations children (Regensburg) and local surveillance data [14,17] season 2023/2024**

**Supplemental** **Figure S3: RSV hospitalisations in Regensburg and Passau since 2018**
